# Homology-based prediction of interactions between proteins using Averaged One-Dependence Estimators

**DOI:** 10.1186/1471-2105-15-213

**Published:** 2014-06-23

**Authors:** Yoichi Murakami, Kenji Mizuguchi

**Affiliations:** 1 Bioinformatics Project, National Institute of Biomedical Innovation, 7-6-8 Saito-Asagi, Ibaraki, Osaka 567-0085, Japan; 2 Graduate School of Information Sciences, Tohoku University, 6-3-09 Aramaki-aza-aoba, Aoba-ku, Sendai, Miyagi 980-8579, Japan

**Keywords:** Prediction of protein-protein interactions, Homology, Machine learning, Averaged One-Dependence Estimators (AODE)

## Abstract

**Background:**

Identification of protein-protein interactions (PPIs) is essential for a better understanding of biological processes, pathways and functions. However, experimental identification of the complete set of PPIs in a cell/organism (“an interactome”) is still a difficult task. To circumvent limitations of current high-throughput experimental techniques, it is necessary to develop high-performance computational methods for predicting PPIs.

**Results:**

In this article, we propose a new computational method to predict interaction between a given pair of protein sequences using features derived from known homologous PPIs. The proposed method is capable of predicting interaction between two proteins (of unknown structure) using Averaged One-Dependence Estimators (AODE) and three features calculated for the protein pair: (a) sequence similarities to a known interacting protein pair (F_Seq_), (b) statistical propensities of domain pairs observed in interacting proteins (F_Dom_) and (c) a sum of edge weights along the shortest path between homologous proteins in a PPI network (F_Net_). Feature vectors were defined to lie in a half-space of the symmetrical high-dimensional feature space to make them independent of the protein order. The predictability of the method was assessed by a 10-fold cross validation on a recently created human PPI dataset with randomly sampled negative data, and the best model achieved an Area Under the Curve of 0.79 (pAUC_0.5%_ = 0.16). In addition, the AODE trained on all three features (named PSOPIA) showed better prediction performance on a separate independent data set than a recently reported homology-based method.

**Conclusions:**

Our results suggest that F_Net_, a feature representing proximity in a known PPI network between two proteins that are homologous to a target protein pair, contributes to the prediction of whether the target proteins interact or not. PSOPIA will help identify novel PPIs and estimate complete PPI networks. The method proposed in this article is freely available on the web at http://mizuguchilab.org/PSOPIA.

## Background

Many biological processes and pathways are mediated by protein-protein interactions (PPIs). Identification of individual PPIs and the whole set of them in a cell/organism (“an interactome”) is, therefore, essential for a better understanding of biological functions of proteins in living cells and elucidating biochemical pathways. Various high-throughput experimental techniques, such as yeast two-hybrid assays and methods based on mass spectrometry, have been used to discover a large number of PPIs in several organisms. Although the amount of interaction data in public PPI databases continues to rise, many of them represent an incomplete interactome, because the available experimental techniques are expensive and can typically identify only a small part of the set of PPIs in specific organisms [1,2].

To circumvent such limitations of the experimental techniques, a number of computational methods have been developed for predicting PPIs based on prior knowledge obtained from known interacting protein sequences and using machine-learning (ML) techniques [3-14]. Efforts have been made to develop methods based only on information about amino acid sequences, for example, by using the number of amino acid triplets in each sequence [6,10,13], a product of signatures defined as a set of subsequences [7], auto-correlation values of seven different physicochemical scales [11,15] and normalized counts of single or pairs of consecutive amino acid residues [12]. These purely sequence-based approaches have reported prediction accuracies of 70-84% on a human data set and about 70% on a yeast data set. Furthermore, information about protein domains has been incorporated in several other methods [16,17]. Although it has been shown to be an informative feature for predicting PPIs [14], methods utilizing domain information alone are not applicable to proteins without domain assignments.

Identifying proteins homologous to a newly determined protein is often attempted to infer the biological functions of the new protein of unknown function, because homologues tend to have similar functions as well as similar three-dimensional structures. This deductive inference has been applied to the identification of PPIs, on the assumption that homologous proteins share similar interaction patterns as well as similar functions [18]. A pair of interacting proteins in one species and their respective orthologs in another species, which are also known to interact with each other, have been traditionally defined as *interaction-orthologs* (interologs) [19,20]. However, this idea can be extended to *interaction-homologs*, because orthologs and paralogs are not always clearly distinguished [18,21].

There have been several computational studies about interologs. For example, Yu *et al.* found that PPIs can be transferred when two pairs of proteins have the geometric mean of the sequence identities >80% or the e-values <10^−70^[20]. Wiles *et al.* predicted PPIs from known interactions in five species and developed InterologFinder, a web server to search for information about predicted as well as experimentally determined PPIs for given proteins of interest [22]. Chen *et al.* developed PPISearch, a web server to search for homologous PPIs given a single protein pair of interest against an integrated database of PPIs in 576 species [18]. Gallone *et al.* developed a Perl module to search for putative PPIs and prioritize them based on interologs [23]. Garcia *et al.* developed BIPS, a web server to predict PPIs based on information about known PPIs in multiple species and additional information about domain interactions and GO annotations. It uses BIANA, an integrated database of PPIs from several repositories [21,24]. In these prediction approaches, collecting as many PPIs as possible in multiple species is an important factor for the reliability of the predicted interactions.

Furthermore, developing a confidence score for PPIs is also key to improving the reliability of the prediction. Most of the previously reported methods used a simple joint sequence identity or e-value for two pairs of interacting proteins [18,20,21], whereas one unified score based on the level of homology, conservation of the interactions across multiple species and the number of supporting experimental types was proposed [22]. These methods are largely dependent on the existence of orthologous or homologous PPIs, i.e., it would be very difficult to detect a novel PPI with no interlogs in an integrated database.

To improve the discrimination power of the homology-based PPI prediction, we here apply Averaged One-Dependence Estimators (AODE; [25]) to this problem. The AODE is an ML algorithm, a variant of the Naïve Bayes classifier (NBC) and it weakens NBC’s independence assumption by allowing a one-dependence. So far, the AODE has been used to combine the outputs of several protein interaction prediction methods; it has been shown to be useful for extracting distinctive information from large imbalanced datasets and it can also be retrained easily and efficiently [26]. Furthermore, it has been reported to be more accurate than NBC, and it can efficiently process a large number of training feature vectors in a high dimensional space without increasing the computational cost significantly [25,27]. In addition, the AODE does not need to select a model and to optimize any parameters. These strengths, therefore, allowed us to train the AODE on massive PPI data collected from several repositories without incurring a large computational cost.

In this study, the AODE is trained using three features: (a) sequence similarities to known interacting proteins (F_Seq_), (b) statistical propensities of domain pairs observed in interacting proteins (F_Dom_) and (c) a sum of edge weights along the shortest path between homologous proteins in a PPI network (F_Net_). The idea of feature (c) is based on the hypothesis that a target protein pair would have more potential to interact if their homologous proteins exist in proximity of each other in a known PPI network. Such a proximal pair, even if not known to interact directly, may form a complex with other proximal proteins or reside in common subcellular locations, thereby increasing the chances of their homologues interacting directly. In a previous study, the topology of a PPI network has been used to predict interactions missing in the network (i.e., those not detected by large-scale experiments), by searching for defective cliques (with a few missing edges) in the PPI network graph [28]. However, this approach can be applied only to proteins with at least one experimentally defined interaction. In addition, the computational cost of this method has been reported to be expensive. Our method, in contrast, searches for a pair of sequences in the graph homologous to the query proteins, which may be unannotated and with no known interactions. Then, a sum of edge weights along the shortest path between them is computed and trained with other features, thus dramatically reducing the computational cost. We demonstrate high predictive performance of the AODE on a recently created human PPI data set with randomly sampled negative data [29], which had been used for benchmarking previously reported sequence-based methods.

## Methods

In this section, we first introduce the data set used for training and testing, and describe three features calculated for a pair of proteins. Next, we describe how to construct a feature vector, dealing with symmetry in the protein order. Then, we describe the AODE for probabilistic classification of protein pairs into interacting (positive) or non-interacting (negative) classes, and introduce prediction accuracy measures to assess prediction models developed and the validation method.

### Preparation of a PPI data set

To train and evaluate AODEs for predicting PPIs, we used two different datasets:

(1) ** Dset1** is a recently created non-redundant human PPI data set (ensuring ≤40% pairwise sequence identity and protein sequence length of >50 amino acids) obtained from the Human Protein Reference Database (HPRD; release 7; [30]), created by [29]. This data set was divided into three independent sets, each of which contained about 2,000 proteins with about 5,000 positive pairs and 2,000,000 negative pairs, i.e., 400 times larger number of non-interacting protein pairs, generated by randomly paring proteins that appeared in the positive pairs and removing real positive pairs. This is a highly imbalanced data set and the classification categories are unequally represented. Park and Marcotte used these subsets to benchmark four different sequence-based PPI prediction methods [29,31] (see Additional file 1: Table S1).

(2) ** Dset2** was constructed to compare prediction performance of the AODE trained on Dset1 with BIPS, a recently developed homology-based prediction server [21]. First, a set of human physical PPIs was obtained from the BioGrid dataset (release 3.2.95, December 2012). Then, from this dataset, we removed PPIs found in the previous BioGrid dataset (release 3.1.93, on October, 2012) compiled after BIPS was released, ensuring that Dset2 includes only recently discovered PPIs. In addition, we used only a set of interacting proteins, each of which was annotated in UniProt [32]. This procedure left a set of 4.430 PPIs. Finally, negative PPI pairs 400 times larger in number than the positives ones were generated in a manner similar to that of Dset1.

### Homology-based features for a pair of proteins

**Figure 1:**
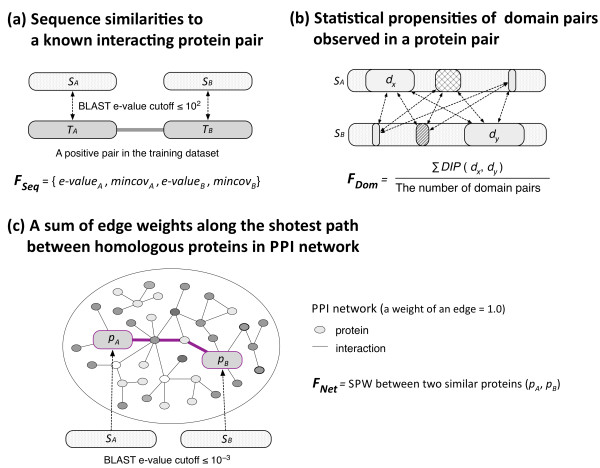
**Three homology-based features used for training AODEs. (a)** A feature set of sequence similarities to known interacting proteins (*F*_*Seq*_ = {*e-value*_*A*_*, mincov*_*A*_, *e-value*_*B*_, *mincov*_*B*_}). For a target pair (*S*_*A*_, *S*_*B*_), the interacting pair (*T*_*A*_, *T*_*B*_) with the smallest value of √(*e-value*_*A*_^2^ + *e-value*_*B*_^2^), where *e-value*_*x*_ is a BLAST *e-value* between *S*_*x*_ and *T*_*x*_ and *x* is either A or B, is selected, and then the minimum coverage (*mincov*) for *S*_*x*_ and *T*_*x*_ is calculated as (the number of positive matches)/(the length of the longer sequence). If no known homologous interacting pair is found, an *e-value* of 10^2^ and a *mincov* of 0 are assigned to F_Seq_. **(b)** Statistical propensities of domain pairs observed in interacting proteins (F_Dom_). A sum of the interaction propensities for all possible Pfam domain pairs (*d*_*A*_, *d*_*B*_) appeared in *S*_*x*_ and *T*_*x*_ is calculated (see more details in the text). If not Pfam domain is found, an F_Dom_ value of 0 is given to the target pair. **(c)** A sum of edge weights along the shortest path between homologous proteins (*P*_*A*_, *P*_*B*_) in the PPI network (F_Net_). In this study, we set the default edge weight to be 1.0. If no path is found, an F_Net_ of -1 is given to the target pair.

The following three features were calculated for a pair of proteins (*S*_*A*_, *S*_*B*_);

(a) ** Sequence similarities to known interacting proteins (F**_Seq_): Known interacting pairs with sequence similarity to a target pair (*S*_*A*_, *S*_*B*_) were searched by running BLAST (version 2.2.25+; [33]) against the database created from the sequences in Dset1, with an e-value cutoff of ≤10^2^. (The high e-value cutoff was chosen to allow for partial matches). Then, of these pairs, the interacting pair (*T*_*A*_, *T*_*B*_) with the smallest value of √(*e-value*_*A*_^*2*^ + *e-value*_*B*_^*2*^) was selected, where *e-value*_*x*_ is the BLAST e-value between *S*_*x*_ and *T*_*x*_ and *x* is either A or B. The minimum coverage (*mincov*) for *S*_*x*_ and *T*_*x*_ was also calculated as the number of positive matches (i.e., alignment positions with a positive BLOSUM62 score [34]) divided by the length of the longer sequence. These two BLAST e-values and two minimum coverage values, (*e-value*_*A*_, *mincov*_*A*_) for *S*_*A*_ and (*e-value*_*B*_, *mincov*_*B*_) for *S*_*B*_, were used as features for training (Figure 1-a). If no known homologous interacting pair was found, an e-value of 10^2^ and a *mincov* of 0 were assigned to F_Seq_.

(b) **Statistical propensities of domain pairs observed in interacting proteins (F**_Dom_): Each sequence in Dset1 was scanned against Pfam-A (release 25.0; Pfam-A.hmm; [35]), and the number of Pfam domain pairs (*d*_*A*_, *d*_*B*_) that appeared in either positive or negative pairs was counted. Knowledge-based interaction propensities for Pfam domain pairs were calculated as:

$$\mathrm{propensity}\left(d_{A},d_{B}\right)=\log\left(\frac{F(+,d_{A},d_{B}/\sum_{x,y\in D}F\left(+,d_{x},d_{y}\right)}{F(-,d_{A},d_{B}/\sum_{x,y\in D}F\left(-,d_{x},d_{y}\right)}\right)$$

where *F*(*c*, *d*_*A*_, *d*_*B*_) is the frequency of a domain pair (*d*_*A*_, *d*_*B*_) observed in protein pairs belonging to class *c* (+; positive, −; negative), and *D* is a set of all Pfam domains observed in Dset1. For each target protein pair, a sum of the interaction propensities for all possible Pfam domain pairs was obtained and divided by the number of the domain pairs. If no Pfam domain was found in *S*_*A*_ and/or *S*_*B*_, an F_Dom_ value of 0 was given to the target pair (Figure 1-b).

(c) **A sum of edge weights along the shortest path between homologous proteins in the PPI network (F**_Net_): BLAST hits (with an e-value cutoff ≤10^−3^) for each sequence in a target pair (*S*_*A*_, *S*_*B*_) were collected from the database created from Dset1. Then, for each possible pair of hits (*p*_*A*_, *p*_*B*_), where *p*_*A*_ and *p*_*B*_ were among the hits for *S*_*A*_ and *S*_*B*_, respectively, a sum of edge weights along the shortest path (the shortest path weight; SPW) was calculated. In this study, we set the default edge weight to be 1.0. The shortest path between *p*_*A*_ and *p*_*B*_ was calculated using Dijkstra’s shortest path algorithm implemented in the Boost::Graph perl module (version 1.4; downloaded from http://search.cpan.org/~dburdick/Boost-Graph/), which is a perl interface to the Boost-Graph C++ libraries (release 1.47.0; downloaded from http://www.boost.org/). The lowest SPW was used as a feature for training. If no SPW was defined for any of the pairs (*p*_*A*_, *p*_*B*_), an F_Net_ value of −1 was given to the target pair (Figure 1-c).

### Constructing a feature vector

**Figure 2:**
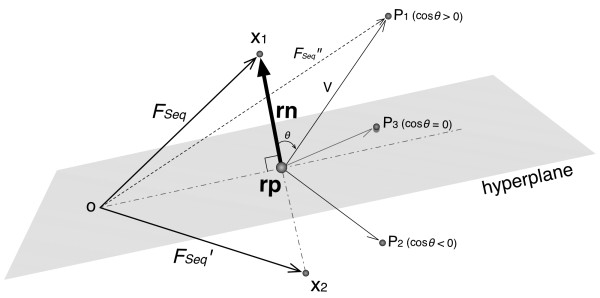
**Selection of feature vectors in the half-space defined by X1.** To define a FV uniquely, an arbitrary pair of proteins whose F_Seq_ and F_Seq_′ values corresponded to points *X*_*1*_ and *X*_*2*_ in the high-dimensional feature space is first chosen. For a given pair of proteins, its FV, F_Seq_′′, was defined by choosing point *P*_*1*_ such that *cos* θ = *rn*⋅*V*/|*rn*||*V*| > 0, where *rn* is a reference normal vector from the midpoint between *X*_*1*_ and *X*_*2*_ (*rp*) to *X*_*1*_, *V* is a vector from *rp* to *P*_*1*_, *rn*⋅*V* is the inner product of *rn* and *V* and |*rn*| and |*V*| are the length of *rn* and *V*, respectively. If *cos* θ = 0, one of the two possibilities was arbitrarily selected.

For each target protein pair, three sequence features described above were computed and converted into a feature vector (FV) = {F_Seq_, F_Dom_, F_Net_}. However, at least two feature vectors can be constructed for F_Seq_, depending on the order of the two protein, i.e., F_Seq_ = {*e-value*_*A*_, *mincov*_*A*_, *e-value*_*B*_, *mincov*_*B*_} and F_Seq_′ = {*e-value*_*B*_, *mincov*_*B*_, *e-value*_*A*_, *mincov*_*A*_}, and in general, F_Seq_′ ≠ F_Seq_. To define a FV uniquely, we first chose an arbitrary pair of proteins whose F_Seq_ and F_Seq_′ values corresponded to points *X*_*1*_ and *X*_*2*_ in the feature space (Figure 2). These points are symmetrically arranged in the four-dimensional feature space separated by a hyperplane. Of the two possible values for any protein pair, we decided to take the one corresponding to a point on the same side of the hyperplane as *X*_*1*_ and denoted this value as F_Seq_". More precisely, for a given pair of proteins, F_Seq_" was defined by the point *P*_*1*_ that had *cos* θ = *rn*∙*V*/|*rn*||*V*| > 0, where *rn* is a reference normal vector from the midpoint (*rp*) between *X*_*1*_ and *X*_*2*_ to *X*_*1*_, *V* is a vector from *rp* to *P*_*1*_, *rn*∙*V* is the inner product of *rn* and *V* and |*rn*| and |*V*| are the lengths of *rn* and *V*, respectively. If *cos* θ = 0, one of the two possibilities was arbitrarily selected as F_Seq_". Finally, a unique FV was constructed as {F_Seq_", F_Dom_, F_Net_}.

After the construction of FVs, feature values for *i*-th feature of the FVs used for training were discretized using the entropy-based discretization method [36]. The optimized intervals (split points), the number of which varied with each feature, were then applied to the construction of FVs for testing.

### Averaged One-Dependence Estimator (AODE)

**Figure 3:**
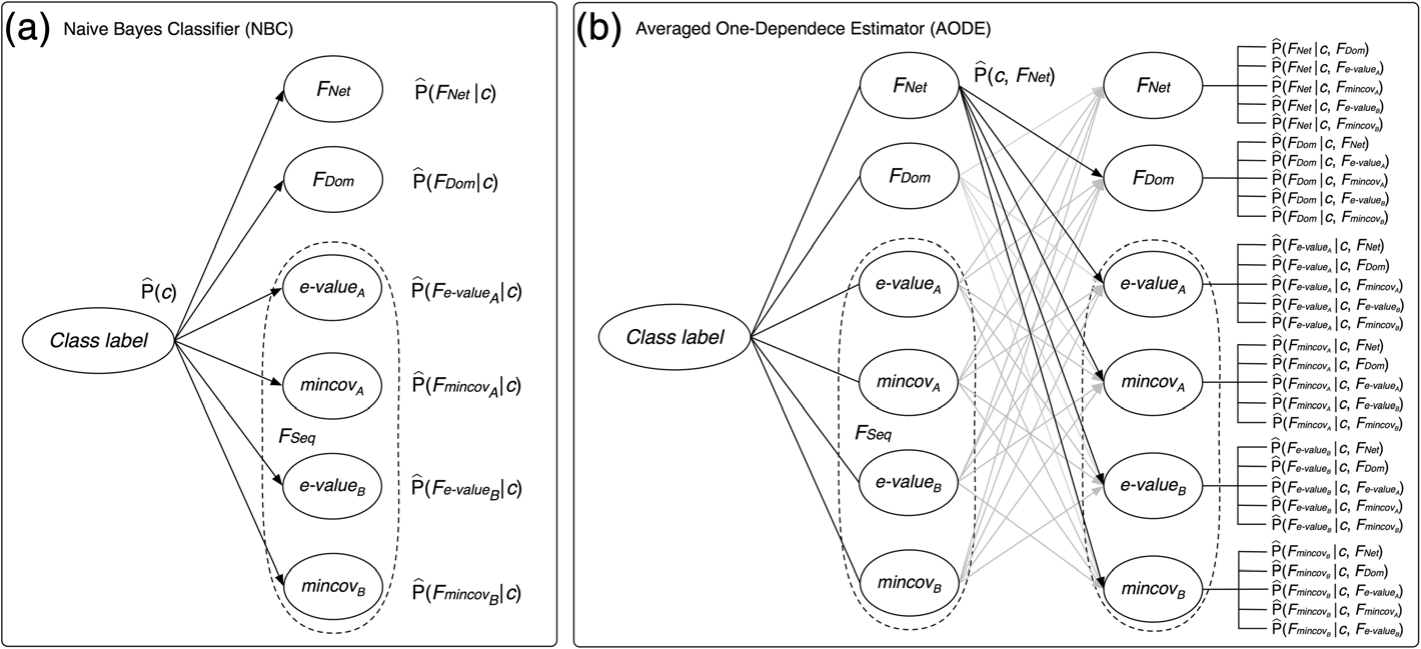
**A probabilistic graphical model of the AODE.** The AODE weakens the NBC’s independence assumption, of which the dependence structure is shown in **(a)**, by allowing a one-dependence as shown in **(b)**. $\hat{P}\left(c\right)$ is the probability of each class label *c* ∈ {*interaction* or *non-interaction*}, $\hat{P}\left(f_{i}|c\right)$, such as $\hat{P}\left(F_{\mathrm{Net}}|c\right)$, is the conditional probability of the *i*-th feature (*f*_*i*_) given *c*, $\hat{P}\left(c,f_{i}\right)$, such as $\hat{P}\left(c,F_{\mathrm{Net}}\right)$, is the joint probability of *c* and *f*_*j*_ and also $\hat{P}\left(f_{j}|c,f_{i}\right)$, such as $\hat{P}\left(F_{\mathrm{Net}}|c,F_{\mathrm{Dom}}\right)$, is the conditional probability of the *j*-th feature (*f*_*j*_) given *c* and *f*_*i*_

The AODE weakens NBC’s independence assumption by allowing a one-dependence, i.e., allowing each feature to depend on another single feature (Figure 3), and it averages the predictions of all one-dependence estimators (ODEs) in each class [25]. The AODE estimates the probability of the positive class (+) given a specified set of features {*f*_*1*_, *f*_*2*_,…, *f*_*n*_}, and is calculated as:

$$\hat{P}\left(+|f_{1},f_{2},\ldots ,f_{n}\right)=\frac{\sum_{i=1}^{n}\hat{P}\left(+,f_{i}\right)\prod_{j=1\wedge i\neq j}^{n}\hat{P}\left(f_{j}|+,f_{i}\right)}{\sum_{c\in \left\{+,-\right\}}\sum_{i=1}^{n}\hat{P}\left(c,f_{i}\right)\prod_{j=1\wedge i\neq j}^{n}\hat{P}\left(f_{j}|c,f_{i}\right)}$$

Here the base probabilities $\hat{P}\left(c,f_{i}\right)$ and $\hat{P}\left(c,f_{i},f_{j}\right)$ were estimated with the Laplace smoothing as:

$$\begin{array}{c} \hat{P}\left(c,f\right)=\frac{F\left(c,f_{i}\right)+1}{m_{i}+kv_{i}}\\ \;\hat{P}\left(c,f_{i},f_{j}\right)=\frac{F\left(c,f_{i},f_{j}\right)+1}{m_{\mathrm{ij}}+kv_{i}v_{j}} \end{array}$$

where F(∙) is the frequency with which a combination of terms appeared in the training FVs, *m*_*i*_ is the number of training FVs for which the *i*-th feature were known, *m*_*ij*_ is the number of training FVs for which the *i*-th and *j*-th features were known, *c* is a class label out of a total of *k* (=2) classes, and *v*_*i*_ and *v*_*j*_ are the number of discrete partitions for the *i*-th and *j*-th features, respectively. Then, the conditional probability $\hat{P}\left(f_{j}|c,f\right)$ was estimated as:

$$\hat{P}\left(f_{j}|c,f_{i}\right)=\frac{\hat{P}\left(c,f_{i},f_{j}\right)}{\hat{P}\left(c,f_{i}\right)}$$

A probabilistic graphical model of the AODE modeled in this study is shown in Figure 3.

**Figure 4:**
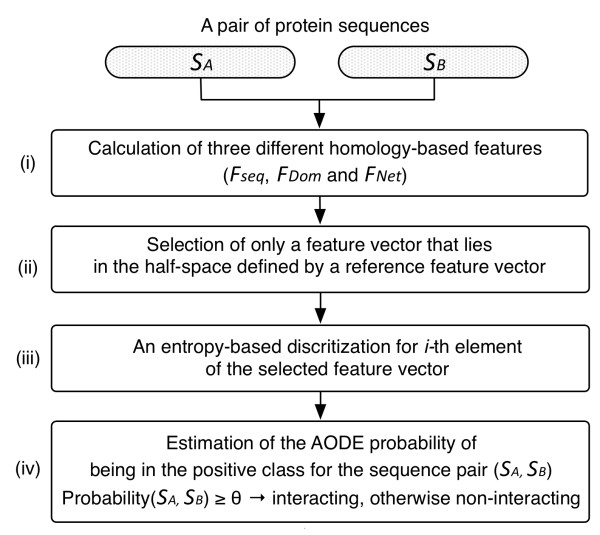
**A schematic diagram to represent the procedure for the proposed method.** (i) Three different homology-based features (F_Seq_, F_Dom_ and F_Net_) for a protein sequence pair are calculated and converted to a FV. (ii) Only the FV that lies in the half-space defined by a reference feature vector is selected (see Figure 2). (iii) A feature value in the *i*-th element of the selected FV is discretized using the entropy-based discretization method [36]. (iv) The probability of being in the positive class for the target pair is estimated using the AODE, and if the probability is greater than or equal to a threshold, the target pair is predicted to be interacting, otherwise non-interacting.

If the probability is greater than or equal to a threshold, the target pair is predicted to be interacting, otherwise non-interacting. A schematic diagram of the prediction procedure is summarized in Figure 4.

### Evaluation measures and validation

Performances of AODEs were estimated by the Area Under the Curve (AUC), which gives an AUC = 1.0 for a perfect model and gives an AUC = 0.5 for a random model for which a Receiver Operating Characteristic (ROC) curve is drawn as a diagonal line. A ROC curve is most often used for model comparison and is represented by plotting sensitivity (true positive rate; TPR, or recall) against 1.0 – specificity (false positive rate; FPR). Sensitivity (recall) measures the proportion of the known positive pairs that are correctly predicted as interacting and is defined as TP/(TP + FN), and specificity measures the proportion of the known negative pairs that are correctly predicted as non-interacting and is defined as TN/(TN + FP), where TP is the number of true positives (i.e., known positive pairs correctly predicted as interacting), FP is the number of false positives (i.e., known negative pairs incorrectly predicted as interacting), TN is the number of true negatives (i.e., known negative pairs correctly predicted as non-interacting), and FN is the number of false negatives (i.e., known positive pair incorrectly predicted as non-interacting). The AUC is known to be insensitive to imbalanced data [37] and it would be a reliable measure for the prediction performance. In addition, performances of AODEs were also estimated by a normalized partial AUC up to the FPR ≤ *x*% (pAUC *x*%), following [6] and [14]. We set *x* to be 0.5. A prediction model with a high pAUC can predict more true positives with few FPs, so such a model is known to be most useful for users to identify PPIs from the top-ranked predictions [6].

Furthermore, we used two other common measures, MCC (Mathew’s correlation coefficient; [38]) and the *F*-measure [39]. MCC indicates the degree of the correlation between the actual and predicted classes of the protein pair, and its values range between 1 where all the predictions are correct, and −1 where none are correct. MCC is defined as (TP × TN − FP × FN)/√(TP + FP) × (TP + FN) × (TN + FP) × (TN + FN). The *F*-measure combines precision and recall into their harmonic mean, and is defined as 2 × precision × recall/(precision + recall), where precision is defined as TP/(TP + FP) and measures the proportion of the positive pairs correctly predicted as interacting.

To evaluate the prediction performance of each AODE, a 10-fold cross validation (CV) was carried out. In the 10-fold CV, a data set was divided into 10 subsets, and each subset was used as a testing set and the remaining subsets were used as a training set. This process was repeated 10 times, and then the prediction performances were averaged over all the test results.

## Results

In this section, we first assess critically the AODE models based on three homology-based features encoded in a single feature vector. We then demonstrate high predictive performance of our proposed method using a large, human PPI data set compiling recently identified interactions.

### Can proximity between homologous proteins in a PPI network contribute to predictions?

We hypothesized that two proteins would have more potential to interact, if their homologous proteins exist in proximity of each other in a known PPI network. Such a proximal pair, even if not known to interact directly, may form a complex with other proximal proteins or reside in common subcellular locations, thereby increasing the chances of their homologues interacting directly. To confirm our hypothesis, we divided Dset1 into 10 subsets, treated each subset as a test set and constructed a PPI network from the remaining subsets. For each pair in the test set, we identified homologous protein pairs (with a BLAST e-value cut-off ≤10^−3^) and obtained the smallest SPW (a sum of edge weights along the shortest path; see METHODS) in the PPI network. In this study, an edge weight of 1.0 was used as a default weight value. This process was repeated 10 times, and the average number of protein pairs with a given SPW was counted.

**Figure 5:**
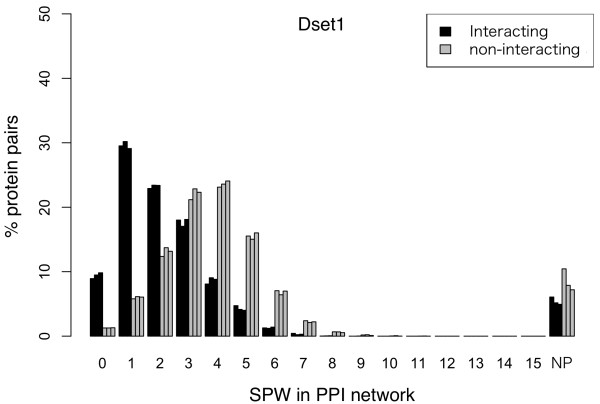
**The percentage of protein pairs with different SPWs in the PPI network generated from Dset1.** An SPW of 0 means a known interaction of a homologous protein with itself and that of 1.0 means a known interaction between a homologous protein pair. Also, a homologous protein pair indirectly linked by *n* proteins has an SPW of *n* + 1.0. NP (No Path) indicates that there was no path between two homologous proteins.

Figure 5 shows the percentage of protein pairs with different SPWs. Note that, in this figure, an SPW of 0 means a known interaction of a homologous protein with itself and that of 1.0 means a known interaction between a homologous protein pair. Also, a homologous protein pair indirectly linked by *n* proteins has an SPW of *n* + 1.0. In consequence, the percentage of positive pairs with an SPW ≤1.0 was about five times lager than that of negative pairs (39% vs 7%). That of positive pairs with an SPW of 2.0 (about 23.2%) was about 10 percentage point lager than that of negative pairs (about 13.1%). Furthermore, a large proportion of positive pairs had SPWs of ≤2.0 (on average, 62.3%), compared to a relatively small proportion of negative pairs (on average, 20.4%). We, therefore, concluded that proximity between homologous proteins in a PPI network could contribute to a discrimination of positive and negative pairs in the PPI prediction, especially, in SPWs ≤2.0. Then, the SPW was chosen as a feature for training the AODE and denoted by F_Net_.

### Prediction performance of AODEs

The AODE was trained and tested on Dset1, a highly imbalanced data set [29]. For a pair of proteins, three different homology-based features, F_Seq_, F_Dom_ and F_Net_, were computed (see Methods). The order of two proteins to define F_Seq_ was determined by selecting its corresponding point in the half-space of the four-dimensional feature space. Then, a set of FVs was constructed to build the AODEs. In addition, all the feature values of a FV were discretized using the entropy-based discretization method [36]. (See Methods and Figures 1, 2, 3 and 4 for more details). In order to assess the predictability of each feature and create the best AODE model, all possible combinations of the three features were examined, where each AODE was evaluated in a 10-fold CV on each independent set of Dset1. In each round of the CV, the AODE was tested on each subset of the independent set, i.e., about 500 positive pairs and 200,000 negative pairs, after trained on the remaining subsets, i.e., about 4,500 positive pairs and 1,800,000 negative pairs. Table 1 shows the prediction performances of different AODEs and, for comparison purposes, those of different NBCs, for different combinations of heterogeneous features. The probability model of the NBC has been introduced in our previous paper [40]. Furthermore, for reference, we included previously reported performances of four different methods benchmarked on Dset1 [29] (Additional file 1: Table S1). While all these methods take protein sequences as input, a direct comparison of the performance is difficult, because our proposed method is based on heterogeneous input features, in contrast to the reported, purely sequence-based methods of M1 ~ M4, which do not use homologous protein sequences explicitly (see Additional file 1: Table S1 for more details of these methods).

**Table 1:** Performances of AODEs and NBCs trained on Dset1

	**Method**	**AODE**		**NBC**		***p*-value**
	**Performance measure**	**AUC**	**pAUC**_**0.5%**_	**AUC**	**pAUC**_**0.5%**_	
I	F_Seq_	0.69 ± 0.01	0.15	0.69 ± 0.01	0.15	0.734
II	F_Dom_	0.57 ± 0.01	0.07	0.57 ± 0.01	0.07	1.0
III	F_Net_	0.77 ± 0.01	0.02	0.77 ± 0.01	0.02	1.0
IV	F_Seq_ + F_Dom_	0.71 ± 0.01	0.16	0.70 ± 0.01	0.16	0.077
V	F_Seq_ + F_Net_	0.79 ± 0.01	0.15	0.77 ± 0.01	0.15	2.8e-08
VI	F_Dom_ + F_Net_	0.79 ± 0.01	0.09	0.77 ± 0.01	0.09	2.7e-08
VII	F_Seq_ + F_Dom_ + F_Net_	**0.79 ± 0.01**	**0.16**	0.77 ± 0.01	0.16	3.9e-14

The AUC and the pAUC_0.5%_ values calculated with 10-fold CV on Dset1 are shown. AUC values given are the mean ± standard deviation. P-values are calculated.

**Figure 6:**
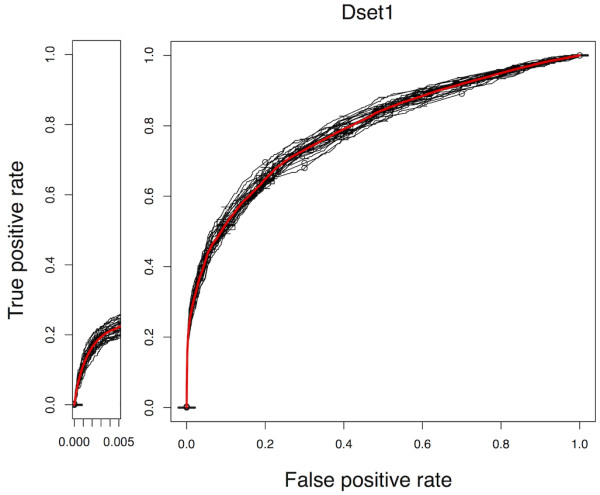
**ROC curves for the AODEs trained with all three features in 10- fold CV.** The ROC curves for the AODE-VII are shown, as well as the averaged ROC curve (in red). This model achieved an AUC of 0.79 and a pAUC_0.5%_ of 0.16% on average, in each round of 10-fold CV on each of the three independent data sets in Dset1.

Of AODEs-I ~ III based on a single feature, AODE-I achieved the highest pAUC_0.5%_ of 0.15 (AUC = 0.69), and AODE-III achieved the highest AUC of 0.77 (pAUC_0.5%_ = 0.02). Of AODE-IV ~ VI, which were created by integrating two features, AODE-VI (based on F_Dom_ and F_Net_) achieved the highest AUC of 0.79. Although AODE-II (based on only F_Dom_) gave the lowest AUC = 0.57 (pAUC_0.5%_ = 0.07) of all three single-feature-based AODEs, integrating F_Dom_ with F_Seq_ or F_Net_ improved both AUC and pAUC_0.5%_, as shown in AODE-IV (AUC = 0.71, pAUC_0.5%_ = 0.16) and AODE-VI (AUC = 0.79, pAUC_0.5%_ = 0.09). AODE-VII integrated all three features and it achieved the highest performance in this CV, in terms of both AUC (0.79) and pAUC_0.5%_ (0.16). In comparison with NBC, AODEs-II and III were identical models to NBCs-II and III, respectively, and no performance difference was observed for methods I and IV. However, including a single dependency with F_Net_ achieved small but statistically significant improvements over NBC. (The p-values from the *t*-test (assuming equal variances) for comparison of AUC values of those methods, i.e., V, VI and VII, were all < 10e-7). While pAUC_0.5%_ values of AODE-VII and NBC-VII were comparable (*p*-value = 0.603), pAUC values up to FPR ≤10%, i.e., pAUC_10%_, were 0.40 for AODE and 0.37 for NBC, respectively (*p*-value = 6.4e-08). These results indicate that making a weaker dependence between features can contribute to the improvement of performance, also in the higher specificity range. Furthermore, AODE-VII outperformed all four previously reported methods in terms of AUC. (As mentioned above, the comparison should be taken with caution and note that pAUCs for M1 ~ M4 were not given in [29]). ROC curves for AODE-VII on Dset1 are shown in Figure 6. In conclusion, AODE-VII achieved the highest performance on Dset1 and thus, it was selected as the best AODE and named PSOPIA (Prediction Server Of Protein-protein InterActions).

### Evaluation of PSOPIA using an independent data set

In order to evaluate our proposed method further, we compared PSOPIA (AODE-VII) with BIPS, a recently developed prediction server based on homologues of two interacting proteins [21]. Because BIPS is based on large, up-to-date PPI data, integrated from several PPI databases by using the BIANA software framework [24], it is considered to have advantages over other similar methods in retrieving homologous PPIs [18,22]. In addition, BIPS can use heterogeneous information similar to PSOPIA for filtering out prediction results, such as information about domain-domain interactions (DDIs) in iPfam [41] and 3DID [42] and annotations from UniProt [32] and GO [43], as well as BLAST-based sequence similarities to a known interacting protein pair. For these reasons, we evaluated the predictability of both PSOPIA and BIPS on Dset2, a data set, which was compiled from a recent release of the BioGrid database and which included only the PPIs identified after BIPS was developed and Dset1 was created (see Methods).

PSOPIA was retrained on the whole of Dset1 and a sequence database used for BLAST was formatted with all the sequences in Dset1. A threshold value of 0.293 was chosen, because it gave the highest *F*-measure (0.160) in the 10-fold CV on Dset1 (recall = 15.5%, precision = 17.0%, specificity = 99.8%, MCC =0.160). For BIPS, since we were unable to optimize the parameters, we used the default values by the web server: joint identities (the geometric mean of individual BLAST sequence identities) ≥ 80%, joint e-values (the geometric mean of individual BLAST e-values) ≥ 1.0 × e^−10^ and template sequence coverage ≥ 80% (see [21] for more details of these parameters). In addition to the default “filter by template interactions”, we also examined two additional filtering conditions: information about DDIs in iPfam or 3DID, and GO annotations (biological process, cellular component or molecular function). The BIPS server accepts sequences of interest or a list of protein identifiers, evaluates potential interactions between all possible sequence pairs and reports only likely (high-scoring) interactions. Therefore, we submitted all the unique sequences in Dset2 to the BIPS server, retrieved the results and defined all the reported pairs to be positive predictions (interacting) and all non-reported pairs to be negative predictions (non-interacting). If a positively predicted pair was found in either the positive or the negative set of Dset2, it was regarded as a true positive or a false positive, respectively. If a negatively predicted pair was found in either the positive or the negative set of Dset2, it was regarded as a false negative or a true negative, respectively. All the other predicted interactions were ignored. In this comparison, we aimed to evaluate the true predictability of these methods, i.e., whether they can predict novel PPIs that have never been observed before, not the data search capability to identify already known PPIs in a database. Thus, we excluded from the evaluation any protein pair (*S*_*A*_, *S*_*B*_) if either BIPS or PSOPIA detected a known interacting protein pair (*T*_*A*_, *T*_*B*_) in their database (with BLAST e-values of 0 for *S*_*A-*_*T*_*A*_ and *S*_*B*_-*T*_*B*_).

Table 2 shows the prediction performances of PSOPIA and BIPS on Dset2. BIPS predictions using template interactions from only human PPIs (taxonomy ID = 9609) (I-A) achieved an *F*-measure of 0.009 (recall = 0.51%, precision = 2.72%). Adding additional information about DDIs and GO annotations reduced the false positives but also reduced the true positives and did not improve the prediction performance (II, III). Furthermore, the use of template interactions from all species increased the false positive in all three options (I) ~ (III) of the BIPS predictions. On the other hand, PSOPIA achieved a higher *F*-measure of 0.030 (recall = 3.33%, precision = 2.77%) at the chosen threshold of 0.293. In addition, by raising the threshold to 0.67 to obtain the recall value of 0.5 ~ 0.6% (comparable to that of BIPS), PSOPIA achieved much higher precision (13.71%) than BIPS (2.72%). In conclusion, in the benchmarking on Dset2, PSOPIA demonstrated higher predictability than BIPS in terms of the *F*-measure.

**Table 2:** Evaluation of true prediction performance on Dset2

**Method**	**TP**	**FP**	**TN**	**FN**	**Sp (%)**	**Re (%)**	**Pr (%)**	**F**
PSOPIA (θ = 0.293, *the higheset F*)	143	5,026	1,766,423	4,152	99.72	3.33	2.77	0.030
PSOPIA (θ = 0.670)	24	151	1,771,298	4,271	99.99	0.56	13.71	0.012
PSOPIA (θ = 0.890)	4	31	1,771,418	4,291	99.99	0.09	11.43	0.002
**(I) BIPS, only filtered by the template interactions**								
(A) Template: Taxonomy ID = 9609 (human)	19	680	1,765,404	3,710	99.96	0.51	2.72	0.009
(B) Template: all species	19	833	1,765,005	3,705	99.95	0.51	2.23	0.008
**(II) BIPS, filtered by known DDIs (iPfam or 3DID)**								
(A) Template: Taxonomy ID = 9609 (human)	5	60	1,771,096	4,261	99.99	0.12	7,69	0.002
(B) Template: all species	5	72	1,771,059	4,256	99.99	0.12	6.49	0.002
**(III) BIPS, filtered by known DDIs (iPfam or 3DID) and GO; biological process, cellular component or molecular function**								
(A) Template: Taxonomy ID = 9609 (human)	3	47	1,771,245	4,284	99.99	0.07	6.00	0.001
(B) Template: all species	3	56	1,771,216	4,280	99.99	0.07	5.08	0.001

For PSOPIA trained on Dset1 (a data set independent of Dset2), the best threshold value, 0.995, which gave the highest F-measure in the 10-fold CV, was used to classify a pair of proteins as interacting or non-interacting. For BIPS, the default values in homologue conditions were used: joint identities ≥ 80%, joint e-values ≥ 1.0 × e^−10^, and template sequence coverage ≥ 80% (see [21] for more details of these parameters). In addition to the filtering by the template interactions only (I), two additional filters were applied: (II) filtered by known DDIs in iPfam or 3DID and (III) filtered by known DDIs and GO annotations (biological process, cellular component or molecular function). Furthermore, two template interactions, (A) only from human (taxonomy ID = 9609) and (B) from all species, were also considered.

## Discussion

We have proposed a new AODE-based method for predicting PPIs based on known homologous PPIs by using three different features, F_Seq_, F_Dom_ and F_Net_. In constructing Dset1 [29] used for training and testing the AODEs, randomly sampled protein pairs that had not been known to interact with each other were used as a negative data set, because of the limited availability of high-quality negative PPI data, either manually curated or experimentally determined (for example, only 1,892 negative PPIs constructed with 1,257 proteins in the negatome database [44]). In reality the number of negative PPIs should be much larger than that of positive PPIs [29,31] and therefore, we trained and evaluated the AODEs on a data set with a large number of negative data. The AODEs were able to deal with this large and imbalanced PPI dataset effectively and they were easily trained within several CPU minutes.

In order to deal with symmetry in the protein order and allow the concatenation of a set of features for individual proteins in a FV, several kernels have been developed in sequence-based methods using a support vector machine (SVM) [6,7,10]. In this study, we proposed a simple geometric selection of FVs in a half space of the symmetrical FV space. Although no comparison can be made between these two approaches, our FV selection method is simple and can be incorporated in any ML method.

The predictability of the AODEs, which include a single dependency between the features, was illustrated in a 10-fold CV on Dset1, and then the AODE trained using all three features, named PSOPIA, achieved the highest performance in terms of both AUC (0.79) and pAUC_0.5%_ (0.16). In comparison with the NBC, which assumes conditional independence of all three features, PSOPIA improved AUC by 0.02 (p-value < 2.8e-08) and pAUC_10%_ by 0.03 (*p*-value = 6.4e-08). We further tested PSOPIA on Dset2, an independent data set, and compared its performance with that of BIPS, a recently reported homology-based method. By excluding the identification of interacting protein pairs already in the database, PSOPIA (threshold = 0.670) achieved higher precision of 13.71% than that of BIPS (2.72%) at a recall level of 0.5 ~ 0.6%, and thus demonstrating higher predictability than BIPS in terms of the *F*-measure. The *F*-measure is generally known as a useful and reliable measure to evaluate different methods that have different trade-off relations between precision and recall.

Further improvements of PSOPIA may be possible by creating a large up-to-date PPI dataset integrated from several databases, because a larger PPI database provides a better chance of detecting known PPIs homologous to a target protein pair. It is still unclear, however, whether we should include cross-species data in such a database. In this study, we evaluated BIPS on Dset2 and showed that the use of interactions from different species did not reduce the false positives. Also, Park [31] and Pitre *et al.*[45] investigated whether interactions for a pair of proteins in a target species can be predicted using a method trained on known PPI data from different species and observed no significant improvements in the performance of the predictors. Thus, it remains to be seen whether the AODE, a probability-based ML method, can improve the prediction performance using interactions from different species as a training dataset. Moreover, it will be worth attempting to change edge weights in a PPI network and distinguish the interaction type, for example, using numerical parameters given by Kerrien *et al.*[46] or similarities in GO annotations [43].

## Conclusions

In this study, we have illustrated that proximity in a known PPI network between two proteins homologous to a target protein pair contributes to the prediction of whether the target proteins interact or not. Then, we have applied this feature F_Net_ to the PPI prediction with two other features, F_Seq_ and F_Dom_. Our best AODE, which achieved an AUC of 0.79 (pAUC_0.5%_ = 0.16) in a 10-fold CV on a highly imbalanced data set, will hopefully contribute to the identification of novel PPIs and the estimation of complete PPI networks. The method proposed in this study is freely available on the web at http://mizuguchilab.org/PSOPIA, and Dset2 used for the evaluation can be downloaded from the same URL.

## Competing interests

The authors declare that they have not competing interests.

## Authors’ contributions

YM developed the methodology and the web server, performed the data analysis and wrote the paper. KM contributed to designing the research, the development and the writing of the manuscript. All authors read and approved the final manuscript.

## Supplementary Material

Additional file 1: Table S1 Performance of four purely sequence-based predictors benchmarked on Dset1, reported by Park [29]. The four methods are; M1: an SVM based on a product of signatures, which encode the sequence information about a protein pair [7], M2: the method based on the co-occurrences of a pair of subsequences appearing in an interacting pair [9,47], M3: an SVM with an S-kernel, which deals with the symmetrical property of PPIs, and was created based on the counts of triplets of amino acids catalogued into seven classes in each sequence [10], M4: an SVM based on auto-correlation values of seven different physicochemical scales calculated for a protein sequence [11]. The pAUC_0.5%_ values for the predictors M1 ~ M4 were not reported. Click here for file
