# Supplementary material for: Homology-based prediction of interactions between proteins using Averaged One-Dependence Estimators
Source: BMC Bioinformatics. 2014 Jun 23;15:213. doi: 10.1186/1471-2105-15-213 (PMC4229973; doi:10.1186/1471-2105-15-213)
Supplement: Additional file 1: Table S1 — Performance of four purely sequence-based predictors benchmarked on Dset1, reported by Park [29]. The four methods are; M1: an SVM based on a product of signatures, which encode the sequence information about a protein pair [7], M2: the method based on the co-occurrences of a pair of subsequences appearing in an interacting pair [9,47], M3: an SVM with an S-kernel, which deals with the symmetrical property of PPIs, and was created based on the counts of triplets of amino acids catalogued into seven classes in each sequence [10], M4: an SVM based on auto-correlation values of seven different physicochemical scales calculated for a protein sequence [11]. The pAUC0.5% values for the predictors M1 ~ M4 were not reported. [file 1471-2105-15-213-S1.docx]

**Supplementary information**

Homology-based prediction of interactions between proteins using Averaged One-Dependence Estimators

Yoichi Murakami^1,2,*^ and Kenji Mizuguchi^1*^
^*^ Corresponding author

^1^ Bioinformatics Project, National Institute of Biomedical Innovation, 7-6-8 Saito-Asagi, Ibaraki, Osaka 567-0085, Japan

^2^ Graduate School of Information Sciences, Tohoku University, 6-3-09 Aramaki-aza-aoba, Aoba-ku, Sendai, Miyagi 980-8579, Japan

**Table S1** Performance of four purely sequence-based predictors benchmarked on Dset1, reported by Park [29]

|  | Method | M1 [7] | M2 [11] | M3 [10] | M4 [47] |  |
| --- | --- | --- | --- | --- | --- | --- |
|  | AUC | 0.72 ± 0.01 | 0.67 ± 0.01 | 0.58 ± 0.01 | 0.72 ± 0.01 |  |

The four methods are; M1: an SVM based on a product of signatures, which encode the sequence information about a protein pair [7], M2: the method based on the co-occurrences of a pair of subsequences appearing in an interacting pair [9,47], M3: an SVM with an S-kernel, which deals with the symmetrical property of PPIs, and was created based on the counts of triplets of amino acids catalogued into seven classes in each sequence [10], M4: an SVM based on auto-correlation values of seven different physicochemical scales calculated for a protein sequence [11]. The pAUC_0.5%_ values for the predictors M1 ~ M4 were not reported.
